# Effects of steam on polysaccharides from *Polygonatum cyrtonema* based on saccharide mapping analysis and pharmacological activity assays

**DOI:** 10.1186/s13020-022-00650-3

**Published:** 2022-08-17

**Authors:** Zherui Chen, Baojie Zhu, Zhixin Chen, Wen Cao, Junqiao Wang, Shaoping Li, Jing Zhao

**Affiliations:** 1 State Key Laboratory of Quality Research in Chinese Medicine, Institute of Chinese Medical Sciences, University of Macau, Macao SAR, China; 2 Joint Laboratory of Chinese Herbal Glycoengineering and Testing Technology, University of Macau, Macao SAR, China

**Keywords:** *Polygonatum cyrtonema*, Polysaccharides, Steam, Chemical composition, Bioactivity, Saccharide mapping

## Abstract

**Background:**

*Polygonatum cyrtonema,* one of origins of Polygonata Rhizoma (*HuangJing* in Chinese), is traditionally steamed repeatedly before being used as herbal medicine in China. However, there has no standard for steaming of *HuangJing*. Therefore, a comprehensive study for effects of steam on polysaccharides from *Polygonatum cyrtonema* based on saccharide mapping, a powerful method developed for polysaccharides analysis, and pharmacological activity are still necessary, which is helpful to explore the effect of steam on the physiochemical and biological activities of its polysaccharides and develop steaming standard of *Polygonatum cyrtonema*.

**Methods:**

To explore the effect of steam on physiochemical and biological activities of *P. cyrtonema* polysaccharides (PCP), six polysaccharides named PCP0, PCP1, PCP2, PCP3, PCP4 and PCP5 were extracted from the herb consecutively steamed for 0–5 times, respectively. Their molecular weight distribution, monosaccharide composition and PACE fingerprints were investigated through HPSEC-MALLS-RID, HPAEC-PAD and saccharide mapping based on polysaccharides analysis by using carbohydrate gel electrophoresis (PACE) and HPTLC, respectively. In addition, their antioxidant ability and immunostimulatory activities on RAW 264.7 cells in terms of NO production and phagocytosis were compared.

**Results:**

Results suggested that molecular weights could be changed during steam, which increased by first steaming and then decreased with further steaming though all polysaccharides’ molecular weights were 10^5^-10^7^ Da. They all showed irregularly spherical conformation in aqueous solution based on AFM imaging. Their monosaccharide composition and PACE fingerprints were significantly different after steaming, i.e., galactose increased while glucose and mannose decreased, and β-1,4-Gal*p* appeared while β-1,4-Man*p* increased, after steaming. Steamed PCP significantly increased scavenging activity against ABTS radicals, while PCP0 had the best immunostimulatory effect on RAW 264.7 in terms of NO production and phagocytosis.

**Conclusions:**

In summary, steam significantly affected the chemical composition and bioactivities of polysaccharides from *P. cyrtonema*. Considering the balance beneficial effects of steaming on antioxidant and immunopotentiation activities of PCP, 2 times of continuous steam is the optimal choice under the given conditions.

**Supplementary Information:**

The online version contains supplementary material available at 10.1186/s13020-022-00650-3.

## Introduction

*Polygonatum cyrtonema* Hua is one of origins of *Polygonata* Rhizoma (*HuangJing* in Chinese), which has a long history of use in China as a tonic herb. It has high medicinal and edible value in medicine and food such as lowering blood sugar [1], lipids [2] and immune potentiation [3]. Polysaccharides are considered as its major active components [4], which showed the effects of anti-oxidation [2, 5–7], anti-aging [8] and immunomodulation [9–14]. According to the theory of traditional Chinese medicine, the processing of Chinese raw materials is usually essential to improve the efficacy or reduce the toxicity of crude drugs [15]. According to *Ben Cao Gang Mu*, *HuangJing* should be processed with “nine steaming and nine drying” (*Jiu-Zheng-Jiu-shai* in Chinese), which means repeatedly steaming and drying until the herb turns black inside before being used as medicine. *Yi Lin Zhuan Yao*, a medical book published in Qing dynasty, recorded that *HuangJing* had the adverse effect of “stinging the throat”. The process of “nine steaming and nine drying” was always used to eliminate or alleviate the adverse reactions, reduce irritation to throat, and improve its efficacy.

However, there had no standardization for steaming of *HuangJing*. In ancient China, people believed the number of steaming times is judged based on its color and macroscopic characters. Recently, changes of small molecular components in *P. sibiricum* (another plant origin of *HuangJing*) during steaming process was reported [16]. Although effects of steam on compositional monosaccharides, molecular weights, antioxidant and immunomodulatory activities of polysaccharides from *HuangJing* have been investigated [17–20], a comprehensive study for effects of steam on polysaccharides from *Polygonatum cyrtonema* based on saccharide mapping, a powerful method developed for polysaccharides analysis, and pharmacological activity is still necessary.

Considering the complexity of polysaccharides, developing a simple and fast qualitative and quantitative method with good accuracy and high specificity has been the key and bottleneck for the quality control of polysaccharides from traditional Chinese medicines. Analytical methods of polysaccharides, including conventional quantitative methods (i.e., colorimetric assays) [21] or recent qualitative methods (i.e., HPLC, GC, FT-IR) [22, 23], show low specificity and poor accuracy for quantification or barely revealed structural information. In view of this, our research group proposed the strategy of saccharide mapping for qualitative analysis and quantitative detection of polysaccharides [24, 25]. For this method, high performance size exclusion chromatography coupled with multi-angle laser light scattering and refractive index detection (HPSEC-MALLS-RID) as one of the most powerful techniques can quickly and accurately determine the content and relative molecular weight of natural polysaccharides and their different components based on their universal refractive index increment (dn/dc) [25]. In addition, saccharides mapping based on polysaccharide analysis using carbohydrate gel-electrophoresis (PACE) is simple, reproducible, high resolution and high throughput. It has been proved to be one of the most effective methods for the quality control of natural resources polysaccharides [24, 26, 27].

In this study, six polysaccharides were extracted and obtained from *P. cyrtonema* consecutively steamed 0–5 times, respectively, and their molecular weights and distributions, UV absorption, saccharide mapping based on polysaccharide analysis by using carbohydrate gel electrophoresis (PACE) and HPTLC, as well as antioxidant and immunostimulatory activities, were investigated and compared. This is the first time to employ saccharide mapping to evaluate the chemical and pharmacological characters of polysaccharides from *P. cyrtonema* with different steam times. Atomic force microscopy was also firstly used to observe the shape and size of these polysaccharides.

## Materials and methods

### Materials and chemicals

Fresh cultivated rhizomes of *P. cyrtonema* were collected from Jinzhai Senfeng Agricultural Technology Development Co., Ltd., Anhui, China. After removing fibrous root, the rhizomes of *P. cyrtonema* were cleaned and then cut into thin slices (3 mm ± 1 mm). Species identification was performed by Professor SP Li, one of the corresponding authors. Voucher specimens of these samples were deposited at the Institute of Chinese Medical Sciences, University of Macau, Macao SAR, China.

ABTS, i.e., 2,2'-Azinobis-(3-ethylbenzthiazoline-6-sulphonate), potassium persulfate and ascorbic acid (≥ 99%) were purchased from International Laboratory (San Bruno, CA), Fluka (Selzer, Germany) and Aladdin (Shanghai, China), respectively. Polygalacturonic acid (PGA), galacturonic acid (GA), konjac glucomannan (KG), dextran (DEX), pectinase (EC 3.2.1.15), endo-1,4-β-D-mannanase (EC 3.2.1.78) and endo-1,4-β-D-galactanase (EC 3.2.1.89) were purchased from Megazyme (Wicklow, Ireland). 8-aminonaphthalene-1,3,6-trisulfonic acid (ANTS) was purchased from Tokyo Chemical Industry (Tokyo, Japan). Griess reagent, lipopolysaccharides (LPS) and fluorescein isothiocyanate-dextran (FITC-Dextran) were purchased from Sigma-Aldrich (St. Louis, MO, USA). Specific limulus test kit for endotoxin detection was purchased from Bioendo Technology, Co., Ltd. (Xiamen, China). Cell counting kit 8 (CCK8) was purchased from MedChemExpress. Phosphate-buffered saline (PBS), Dulbecco’s Modified Eagle Medium (DMEM), fetal bovine serum (FBS), and penicillin/streptomycin (P/S) were purchased from Gibco-Invitrogen (Paisley, Scotland, UK). Nylon membrane filters (0.22/0.45 μm) were purchased from Millipore (Billerica, MA). Deionized water was prepared using a Millipore MilliQ-Plus system (Millipore, Billerica, MA). All the other reagents were of analytical grade.

### Preparation of *P. cyrtonema* and polysaccharides

#### Steam of *P. cyrtonema*

Fresh *P. cyrtonema* slices (dried as PC0) were steamed, over water, in an autoclave at 115 °C for 2 h at pressure of 0.07 MPa with good repeatability. The steamed material dried under vacuum at 60 °C to constant weight and collected as the sample PC1. Then half of PC1 was moistened with deionized water before further steam and dried under mentioned conditions above to obtain PC2. Similarly, PC3, PC4 and PC5 were prepared, respectively. All samples were grounded, and the particle size was 355 μm ± 13 μm.

#### Extraction of polysaccharides

Each sample (PC0-PC5) of 50.0 g was soaked at room temperature for 2 h, followed by hot water extraction (95 °C) for further 2 h with solid-to-liquid ratio of 1:15. The water extracts were precipitated with ethanol at a final concentration of 75% to harvest polysaccharides [28]. The supernatant solution (PCS) and precipitate (PCP) were collected. Then small molecules (less than 3 KDa) mixed in polysaccharides were further removed using ultrafiltration. Finally, the polysaccharides were dried with freeze-drier to obtain PCP0, PCP1, PCP2, PCP3, PCP4 and PCP5, respectively.

### Sample pretreatment of PCP

#### Partial and complete acid hydrolysis of PCP

According to a previously reported method with minor modification [29], PCP solution (2 mg/mL) was treated with trifluoroacetic acid (TFA) at a final concentration of 1.0 mol/L and incubated at 80 °C for 2 h to gather partial acid hydrolysates (PAH). At the same time, the PCP solution (4 mg/mL) of each sample was mixed with an equal volume of 4 mol/L TFA for complete acid hydrolysis at 105 °C for 4 h to gather complete acid hydrolysates (CAH).

#### Enzymatic hydrolysis of PCP

According to literature [30], three enzymes of pectinase, β-1,4-galactanase and β-1,4-mannanase were selected to depolymerize PCP (2 mg/mL) at 40 °C for 12 h. After incubation, enzymes were inactivated at 80 °C for 20 min. For PACE analysis, the enzymatic PCP hydrolysates should be firstly freeze-dried and derivatized with ANTS. While no additional treatment is required for HPTLC and monosaccharides analysis. Polysaccharide standards, including PGA, GA and KG, were treated with those enzymes, respectively, under the same conditions. PCP solution without TFA or enzyme treatment was used as blank control.

### Physicochemical characterization of polysaccharides

#### Molecular weights and chain conformation analysis

The molecular weights and their distribution of PCP were determined by HPSEC-MALLS-RID according to our previous report [28]. HPSEC-MALLS-RID method was composed of multi-angle laser light scattering (MALLS) detectors (Wyatt Technology Co., Santa Barbara, CA, USA), Agilent 1260 series LC/DAD system (Agilent Technologies, Palo Alto, CA, USA) and a refractometer (RID, Optilab rEX, Wyatt Technology Co.) in series at 35 °C. The chromatographic columns were TOSOH gel columns TSK-GEL G5000PWXL (300 mm × 7.8 mm) and TSK-GEL G3000PWXL (300 mm × 7.8 mm), and the mobile phase was 0.9% NaCl solution. The flow rate was 0.5 mL/min with 100 μL injection volume. All samples of PCP were dissolved in mobile phase at 2 mg/mL and filtered through 0.45 μm filter membrane before injection. ASTRA 7.3.2 software was used to process the data.

#### Compositional monosaccharides analysis

The complete acid and pectinase hydrolyzed samples as well as PCS were analyzed by HPAEC-PAD system (Thermo Scientific^™^ Dionex^™^ ICS-5000^+^, Dionex, USA). Ten reference monosaccharides, including Fuc, Ara, Rha, Gal, Glc, Xyl, Man, Fru, GalA and GlcA, were used to calculate the content of each monosaccharide in the samples using one-point calculation of external standard method. All samples were filtered through 0.45 μm membrane before analysis. The mobile phase consisted of 88% deionized water and 12% 10 mM NaOH, running for 22 min at a flow rate of 0.4 mL/min on a CarboPac PA200 (3 mm × 250 mm) analytical column with a system temperature of 25 °C (Additional file  1: Table S1).

#### PACE analysis of partial acid and enzymatic hydrolysates

PACE was performed according to previous report [31]. Briefly, all lyophilized derivatized hydrolysates of PCP were redissolved in isovolumetric urea (6 mol/L), and separated by Mini-Protean Tetra System, a vertical slab gel electrophoresis apparatus from Bio-Rad. Gels were imaged using an In-Genius LHR CCD camera system (Syngene, Cambridge, UK) under UV 365 nm. Quantity-One software (Ver4.6.2, BioRad) and Similarity Evaluation System for Chromatographic Fingerprint of Traditional Chinese Medicine (Matlab version, Ver1.315, developed by the Research Center of Modernization of Chinese Herbal Medicine, Central South University and the Hong Kong Polytechnic University) were used for similarity analysis.

#### HPTLC analysis of complete acid and pectinase hydrolysates

Merck silica gel 60 plates pre-washed with methanol were used for HPTLC analysis. The method was modified according to previous report [31]. In brief, PCP0-PCP5 samples after complete acid or pectinase hydrolysis were prepared into 8 mg/mL, respectively. SP-III electric thin-layer strip sampler (KEZHE SHANGHAI, China) was used for semi-automatic sampling. The bands were 7 mm wide, 5 mm distance, and 10 mm from the bottom edge. Then the plate was firstly developed to 90 mm with 1-butanol/isopropanol/acedic acid/water, 7:5:1:2 (v/v/v/v) as developing reagent at room temperature. Then the plate was dried and placed in the same chamber to develop 95 mm with the same developing reagent as described above. Finally, the developed plates were dried and colorized with aniline-diphenylamine-phosphoric acid solution, 10% sulfuric acid ethanol solution and 0.2% ninhydrin solution, respectively, then heated at 105 °C and photographed under white light.

#### AFM analysis

PCP0-PCP5 (1 mg/mL) were fully dissolved in ultrapure water and diluted to a concentration of 1 × 10^−2^ μg/mL. Using droplet deposition method, pipette 5μL of solution onto the surface of newly cut mica sheet and dry it at room temperature. After the sample dried, AFM measurement was performed using BioScope Resolve AFM (Bruker Co., Santa Barbara, USA), and NanoscopeAnalysis 1.8 software was used for image analysis.

### Antioxidation of PCP against ABTS radicals

In brief, 7 mmol/L ABTS aqueous solution and 2.5 mmol/L potassium persulfate aqueous solution were mixed in a ratio of 1:1, and then stand in the dark for 12 h. This solution was diluted with deionized water to reach a 0.7 ± 0.05 absorbance value at 734 nm and obtained ABTS working solution. After preliminary optimization, series concentrations of PCP0-PCP5 (0.5, 1, 2, 4, 8 mg/mL) and ascorbic acid (0.015, 0.03, 0.06, 0.12, 0.24, 0.5, 1, 2, 4, 8 mg/mL) were used. In a 96-well plate, 200 μL of ABTS working solution and 10 μL of the sample solution were added to each well, and the reaction was kept in the dark for 6 min. Then the absorbance was measured, and the ABTS clearance rate formula was as followed:

C (%) = [1-(A_1_-A_2_)/A_0_] × 100.

C was the clearance rate, A_0_ is the control absorbance, A_1_ is the sample absorbance, and A_2_ was the background absorbance, which was to eliminate the interference of tested solution.

### Effects of PCP on macrophage functions

#### Cell culture

RAW 264.7 cells were purchased from American Type Culture Collection (ATCC, Rockville, MD, USA). Cells were cultured in Dulbecco’s modified eagle medium supplemented with 10% FBS and 1% P/S at 37 °C in a humidified atmosphere of 5% CO_2_.

#### Cytotoxicity assay

RAW 264.7 cells (5 × 10^3^ cells/well) were cultured in 96-well microplates overnight, and then treated with LPS (0.4 μg/mL) and a series of concentrations of PCP for 24 h, respectively. An equal volume of culture medium was used as blank control. Subsequently, the original culture medium was discarded and stained with 100 μL of culture medium containing 10% CCK8 for 4 h in dark. The absorbance values were read at 450 nm and the cell viability was calculated as the ratio of absorbance values between the sample and vehicle control group.

#### Nitric oxide determination

RAW 264.7 cells (5 × 10^4^ cells/well) were seeded in 96-well microplates overnight, and then cells were treated with a series of concentrations of PCP and LPS (0.4 μg/mL) for 24 h, respectively. An equal volume of culture medium was used as vehicle control. Subsequently, 75 μL of supernatants were collected and mixed with an equal volume of modified Griess reagent at room temperature for 15 min. The absorbance was measured at 540 nm. NO production was expressed as the ratio of absorbance values between sample and LPS treated group.

#### Phagocytic activity test

FITC-dextran was used for phagocytic assay. RAW 264.7 cells (1 × 10^5^ cells/well) were cultured in 24-well plates overnight and then incubated with culture medium, LPS (0.4 μg/mL) and a series of concentrations of PCP for 18 h, respectively. Then the cells were treated with FITC-dextran (0.1 mg/mL in culture medium) and incubated at 37 °C for an additional 1 h in dark. After incubation, the cells were collected with cold PBS after washed for three times. BD Accuri^™^ C6 Cytometer (BD Biosciences, San Jose, CA, USA) was used for analysis. The percentage of phagocytosis was expressed as ratio of phagocytic rate between the treatment and control cells.

#### Determination of endotoxin contamination

Endotoxin detection specific limulus test kit was used for avoiding endotoxin contamination. The results showed that PCP0-PCP5 had no endotoxin contamination in the samples.

### Statistical analysis

GraphPad Prism 8.0.2 was used to analyze and process the data. Data were presented as mean ± SEM from at least three independent experiments for each sample. Statistical significance between the experimental groups was determined by Student’s t-test, and *p* values less than 0.05 were considered as statistically significant.

## Results and discussion

### Appearance of *P. cyrtonema* and its polysaccharides

**Fig. 1:**
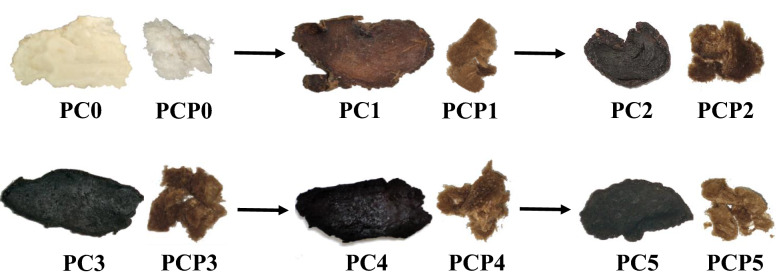
Pictures of *Polygonatum cyrtonema* slices steamed 0–5 times and *P. cyrtonema* polysaccharide powder

After steam treatment, the color of *P. cyrtonema* slices deepened gradually. The raw material (PC0) was milky white, after the first steam the color turned reddish brown with a lighter color in the center (PC1). Then, after the second time of steam treatment, slice PC2 changed to dark brown. Slice PC3 changed into black and began to appear luster, the color of PC4 and PC5 almost no further change compared with PC3. The color of the polysaccharide powders was also changed accordingly, similar to the raw materials (Fig. 1). Traditionally, the number of steam treatment according to the color and macroscopic characters of materials. Actually, the color of PC5, same as those of PC3 and PC4, was black. Therefore, 5 times were selected for steam of *P. cyrtonema*.

### Molecular weight distribution of PCP

**Fig. 2:**
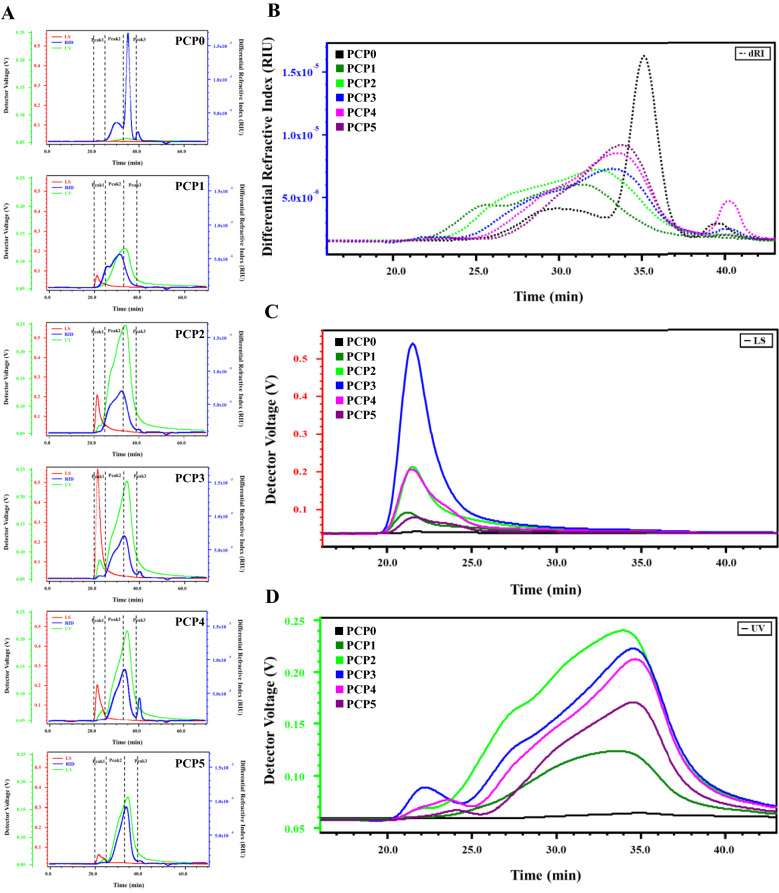
HPSEC-MALLS-RID chromatograms (**A**), comparative chromatograms on RI (**B**), LS (**C**) and UV (**D**) chromatograms of PCP0-PCP5

HPSEC-MALLS-RID is a useful method for determining the absolute molecular weights (Mw), dispersibility index (DPI) and radius of gyrations (Rg) of polysaccharides without standards [32]. Figure 2 showed that HPSEC-MALLS-RID chromatograms of PCP in 0.9% NaCl aqueous solution at 35 °C. The molecular mass distribution of PCP0-PCP5 were summarized in Table 1. To facilitate the comparison of steaming treatment on molecular weight distribution of PCP, the same peak division was made on PCP1-PCP5 according to that of PCP0, as shown in Fig. 2A, which was divided into three peaks. In PCP0, Peak3 had the highest content and a molecular weight of ~ 7.85 × 10^3^ Da, with Mw/Mn of 1.16. After the first steaming treatment, Mw at Peak3 of PCP1 increased dramatically to 1.02 × 10^5^ Da, and gradually decreased with the increase of steaming times (Fig. 2B). This indicated that steam could change the extraction and/or molecular weight distribution of PCP. Similarly, Mw of PCP increased by 247.4% in the first 4 h of steaming compared to the original PCP and decreased with the increasing steam time [17]. In addition, the detection at UV 280 nm showed that PCP0 had a very low signal, but steamed PCP showed obvious UV signals. With the increase of steaming times, UV signal increased firstly, PCP2 to the highest, and then decreased gradually (Fig. 2D). This implied that steaming treatment induced Maillard reaction of polysaccharides in *P. cyrtonema* [33, 34].

**Table 1:** The molecular weight, polydispersity index (Mw/Mn) and contents of PCP0-PCP5

Sample	Peak 1				Peak2				Peak3				Total content %
	Mw, Da (error %)	Mw/Mn (error %)	Rz, nm	Content %	Mw, Da (error %)	Mw/Mn (error %)	Rz, nm	Content%	Mw, Da (error %)	Mw/Mn (error %)	Rz, nm	Content %	
PCP0	1.08 × 10^7^ (± 2.8%)	1.43 (± 5.1%)	56.7 (± 4.2%)	0.1	7.04 × 10^4^ (± 2.4%)	3.64 (± 5.6%)	21.9 (± 18.5%)	20.7	7.85 × 10^3^ (± 2.6%)	1.16 (± 6.4%)	17.9 (± 29.3%)	49.5	70.3
PCP1	1.01 × 10^7^ (± 3.9%)	1.70 (± 6.2%)	65.8 (± 4.2%)	1.4	1.73 × 10^5^ (± 2.8%)	3.55 (± 6.4%)	34.4 (± 9.1%)	48.6	1.02 × 10^5^ (± 3.3%)	1.18 (± 7.2%)	39.2 (± 8.7%)	12.5	62.5
PCP2	5.87 × 10^7^ (± 4.0%)	1.44 (± 5.7%)	52.0 (± 6.4%)	0.7	3.81 × 10^5^ (± 3.6%)	3.15 (± 4.8%)	29.0 (± 15.1%)	54.0	1.46 × 10^5^ (± 4.2%)	1.18 (± 5.3%)	31.5 (± 15.3%)	23.9	78.6
PCP 3	9.28 × 10^7^ (± 4.5%)	1.88 (± 5.4%)	42.3 (± 10.4%)	0.9	7.88 × 10^5^ (± 3.3%)	2.15 (± 5.3%)	37.2 (± 9.2%)	41.3	1.69 × 10^5^ (± 3.7%)	1.18 (± 6.0%)	39.7 (± 9.2%)	28.4	70.6
PCP 4	7.93 × 10^7^ (± 4.6%)	3.84 (± 4.9%)	50.3 (± 7.6%)	0.5	4.08 × 10^5^ (± 4.2%)	1.85 (± 4.1%)	30.7 (± 16.0%)	40.3	7.98 × 10^4^ (± 5.1%)	1.22 (± 4.8%)	33.4 (± 16.6%)	34.5	75.3
PCP 5	2.92 × 10^7^ (± 3.8%)	1.16 (± 4.0%)	44.7 (± 7.9%)	0.3	2.06 × 10^5^ (± 3.6%)	2.59 (± 3.5%)	23.0 (± 24.7%)	32.4	3.64 × 10^4^ (± 4.5%)	1.06 (± 3.5%)	22.2 (± 32.8%)	36.5	69.2

### Monosaccharide composition

After complete acid hydrolysis, compositional monosaccharides of PCP were determined by HPAEC-PAD (Additional file 1: Figure S1). Results showed that PCP without steam treatment was mainly composed of GalA, Man and Glc. After steaming, all PCPs were mainly composed of Gal, Man and GalA, with a small amount of Ara, Rha, Glc and Xyl. The molar ratios of Ara, Rha, Gal, Glc, Xyl, Man, Fru and GalA in PCP0-PCP5 were 21.0:6.7:18.3:56.3:1.0:107.4:14.3:41.1, 9.4:4.0:29.2:4.2:1.0:24.0:0.1:20.0, 7.7:4.9:38.5:3.2:1.0:20.8:0.1:20.0, 4.5:5.6:41.6:2.5:1.0:15.0:0.1:14.6, 2.7:4.9:32.5:2.2:1.0:12.6:0.0:10.6 and 1.6:5.0:29.3:2.3:1.0:12.7:0.0:8.5, respectively (Additional file 1: Table S2). Obviously, the compositional monosaccharides of PCPs changed dramatically after the first steaming. The content of Man and Glc decreased significantly while the content of Gal increased obviously. As the steaming times increased, the content of Man and Glc remained stable starting from PCP3 while Gal still decreased in PCP4 and PCP5. At the same time, GalA and Ara also decreased. According to previous studies [35], PCP mainly contained Man, Glc, Gal and Ara, which was consistent with our results. Interestingly, according to the study by Li et al.[18], Glc and Ara increased with the steam treatment, was not inconsistent with our research results, might attribute to different samples and/or steaming conditions.

Besides, previous studies have shown that PCP contained a lot of fructose [19], while the conventional complete acid hydrolysis could induce fructose loss. In addition, galacturonic acid is easily degraded under acid hydrolysis, which result in wrong results. Therefore, pectinase was also used for hydrolysis and observed the changes of PCP0-PCP5 in monosaccharides. Though incomplete hydrolysis might be obtained, the data should still be very helpful to compare the easily degraded monosaccharides release from PCP0-PCP5 (Additional file 1: Figure S2). The results showed that the molar ratio of Ara, Rha, Gal, Glc, Xyl, Man, Fru and GalA in PCP0-PCP5 was 59.4:7.5:56.3:57.5:1.0:59.7:415.2:263.0, 33.5:8.9:102.7:5.8:1.0:22.9:6.8:150.5, 26.4:9.8:137.5:6.4:1.0:21.0:2.5:105.1, 12.4:10.9:120.5:6.7:1.0:16.5:1.8:84.6, 7.6:10.3:98.7:5.7:1.0:18.0:1.6:63.8 and 3.7:10.4:85.9:3.6:1.0:18.8:0.8:47.3, respectively (Additional file 1: Table S2). It suggested that ratio of Fru decreased dramatically after steam treatment and almost disappear in PCP1-PCP5. But ratio of GalA increased after steaming though its degradation could be found with the steam time increased. This indicated that pectinase hydrolysis was helpful to indicate the change of PCP based on Fru and GalA.

At the same time, we examined changes in Fru and Glc in 75% ethanol supernatant during ethanol precipitation, and the contents of Fru and Glc in the supernatant increased with steaming. The results showed that the composition of polysaccharides changed with the increase of steaming times, and the contents of monosaccharides such as fructose and glucose or oligosaccharides in free state increased. As polysaccharides are relatively stable under the condition of normal temperature, however, under the steaming condition of high pressure and high temperature, various monosaccharide components can undergo dehydration and degradation, and Maillard reaction [34] can occur under the common existence of other components such as amino acids. Polysaccharides are composed of various monosaccharides, which can be changed by degradation and/or reaction during steam processing. The contents of fructose and glucose in PCS suggested that steam could induce degradation of PCP and release free monosaccharides, Fru and Glc, which also might result in low relative ratio of Gal and/or its degradation. The changes of PCP during steam processing need further investigated.

### PACE profiles of PCP

**Fig. 3:**
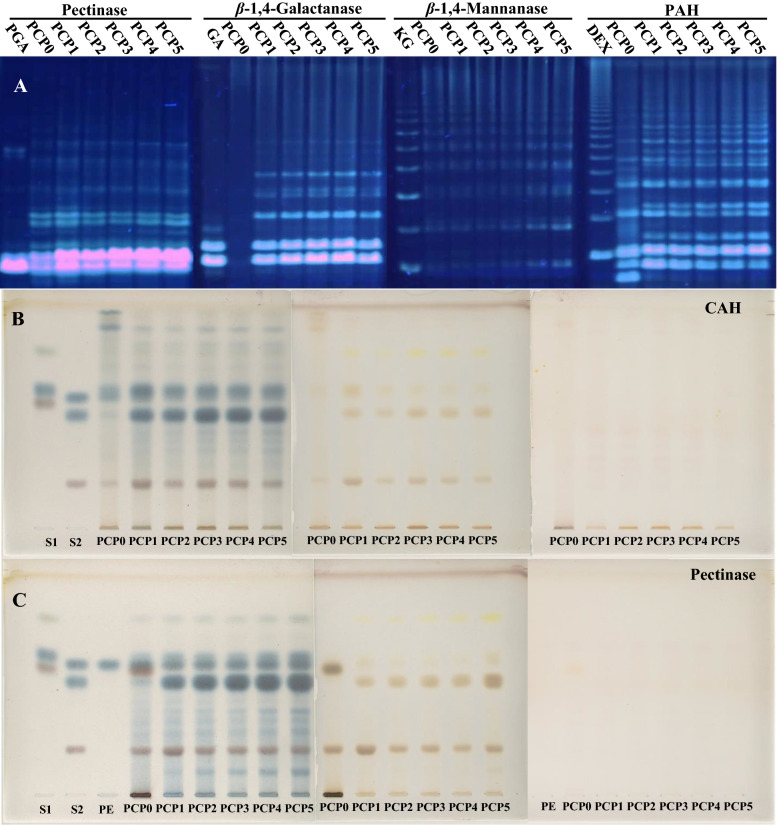
PACE profiles of partial acid hydrolysis (PAH) and enzymatic digestion of PCP (**A**), HPTLC profiles of complete acid hydrolysis (CAH) (**B**) and pectinase hydrolysis (**C**) of PCPs, colorized with aniline-diphenylamine-phosphoric acid solution, sulfuric acid ethanol solution and ninhydrin solution from left to right. PGA is polygalacturonic acid, GA is galacturonic acid, KG is konjac glucomannan, DEX is dextran. S1 is Rha, Man and Fru from top to bottom, S2 is Glc, Gal and GalA from top to bottom respectively, PE is the blank control of pectinase

Saccharide mapping based on PACE has been proven to be one of powerful methods for routine analysis of oligosaccharides derived from polysaccharides [31]. Therefore, both partial acid hydrolysates and enzymatic hydrolysates of PCP were compared using saccharide mapping based on PACE analysis (Fig. 3A), and their similarity was conducted using Quantity-One software and Similarity Evaluation System. Their hydrolysates had high similarity except partial acid and β-1,4-galactanase hydrolysates of PCP0 (Table 2). The fingerprints of β-1,4-galactanase hydrolysates indicated that there was no β-1,4-Gal*p* detected in PCP0, but rich in PCP1-PCP5 after steaming, which was also supported by HPSEC-MALLS-RID and HPAEC chromatograms (Additional file 1: Figure S3 and S4). These finding suggested that steaming could significantly affect some chemical characters of PCP.

**Table2:** The correlation coefficient of PCPs to their simulative mean chromatogram

Samples	The simulative mean chromatograms					
	PACE				HPTLC	
	SMC-PAH	SMC-GA	SMC-MA	SMC-PE	SMC-PE	SMC-CAH
PCP0	1.00	1.00	1.00	1.00	1.00	1.00
PCP1	0.75	0.00	0.81	0.96	0.76	0.51
PCP2	0.76	0.00	0.77	0.96	0.77	0.51
PCP3	0.76	0.00	0.88	0.96	0.77	0.51
PCP4	0.75	0.00	0.89	0.96	0.77	0.51
PCP5	0.72	0.00	0.88	0.96	0.76	0.50

### HPTLC fingerprints of PCP

HPTLC showed the samples of complete acid hydrolysis and pectinase-digested PCP had varied results. According to phenylamine-phosphoric acid coloration, pectinase hydrolysates contained more oligosaccharides than completely hydrolyzed samples (Fig. 3B). Colorization of ninhydrin coloration showed that there almost were no amino acids released from PCP before and after steam (Fig. 3B and C). HPTLC profiles similarity was shown in Table 2, which both pectinase and complete acid hydrolysates of PCP indicated that there was significant difference among samples before and after steam.

### Morphology of PCP

Biological activities of natural polysaccharides are closely related to their chain conformation besides molecular weights [36]. Therefore, it is very important to study the effect of steam on chain conformation of PCP in aqueous solution. The conformation of polysaccharides can be analyzed according to the theory of dilute polymer solution. Generally, the chain conformation of polysaccharides in aqueous solution is determined by the double logarithmic plot of Rg vs the molecular mass of polysaccharides according to Mark-Houwink equation Rg = kMw^ν^ [37]. According to the polymer solution theory, the exponent (*v*) is 0.2–0.4 for branched polymers with a compact helical chain conformation, 0.3 for spheres, 0.5–0.6 for flexible polymers in good solvents, and 0.6–1.0 for semi-flexible chains [38]. According to the calculation results of HPSEC-MALLS/RI, the *v* index of PCP0-PCP5 was concentrated between 0 and 0.3 (Additional file 1: Figure S5). The results showed that PCP0-PCP5 appeared as irregular monodisperse spheres in 0.9% aqueous sodium chloride solution.

**Fig. 4:**
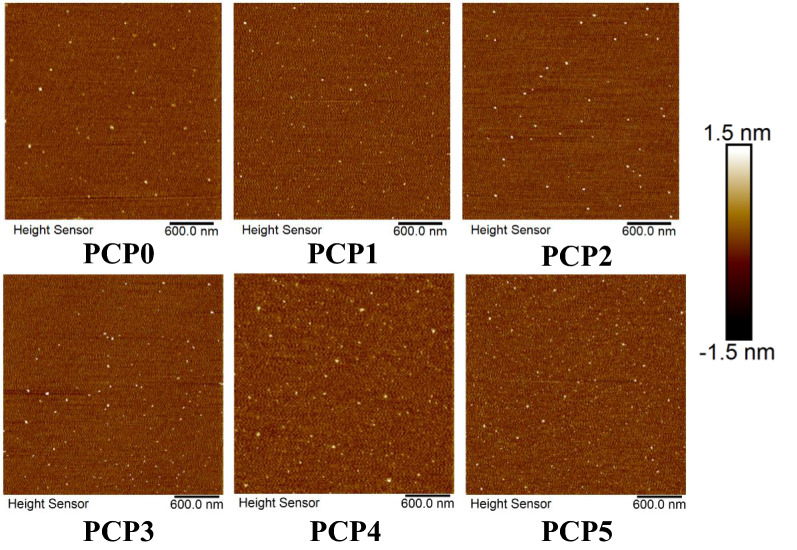
Planar view of 0.01 μg/mL PCP0-PCP5 observed under atomic force microscope

Atomic force microscopy (AFM) has also become a powerful tool to directly characterize the structure and properties of polymers [39]. The planar images with height and diameter (scanned at 3 × 3 μm) of PCP0-PCP5 in aqueous solution obtained by AFM were shown in Fig. 4. An irregular disperse spherical shape of all PCP was observed, consistent with the results of HPSEC-MALLS-RID. Their molecular height was in the range of 1–1.5 nm, and the diameter ranged from 15 to 20 nm. Specifically, with the steaming times increased, the height of PCP increased, and the diameter decreased gradually. There was almost no significant difference after the second steaming, except PCP4 with the height and diameter of 1.4 nm and 20.0 nm, respectively. This is likely to be related to the change of polysaccharides structure and/or fractions caused by steaming.

### ABTS scavenge ability of PCP

**Fig. 5:**
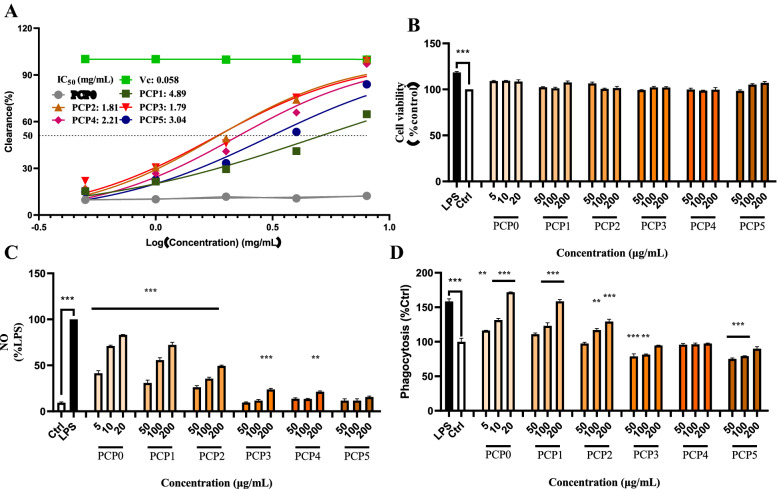
Effects of PCP0-PCP5 on ABTS free radical scavenging activity (**A**), as well as cell viability (**B**), NO production (**C**) and phagocytosis (**D**) of RAW 264.7 macrophages. All values were expressed as mean ± SEM of three independent experiments. *p < 0.05, **p < 0.01, ***p < 0.001 *vs* control group, unmarked results indicate no significant difference *vs* control group

ABTS scavenge ability of PCP was shown in Fig. 5A, and all PCP showed scavenge ability against ABTS radicals in different extents, and PCP0 showed the lowest capacity. Generally, steamed PCP had higher dose-dependent free radical scavenging ability, and IC_50_ values of PCP1-PCP5 were 4.89, 1.81, 1.79, 2.21 and 3.04 mg/mL, respectively. Change of steamed PCP in antioxidant capacity may attribute to Maillard reaction of polysaccharides during steam processing [33], which was supported by UV 280 nm absorption and molecular weights increased after steaming (Fig. 2). Though the ability of PCP scavenging ABTS radicals increased with the number of steaming times [18], IC_50_ showed that antioxidant activity of both PCP2 (1.81 mg/mL) and PCP3 (1.79 mg/mL) reached to the most potent, and then antioxidation decreased with increase of steaming times. Anyway, steaming significantly enhances antioxidant capacity of PCP, which is beneficial to its efficacy in delaying aging [8], lowering blood sugar [6, 40, 41] and regulating blood lipids [2]. The significance of steaming to health beneficial effects of *P. cyrtonema* should be further comprehensively investigated though 2–3 times steaming seemed have good antioxidant activity. Actually, the optimum steaming times could be different based on various steaming conditions and investigated biological activities [17, 18].

### Immunostimulatory activity of PCP

Macrophages play an indispensable role in the innate and adaptive immunity of human body [42]. Studies have shown that high levels of NO are associated with immune responses during antitumor and antiviral processes, which can trigger cell proliferation, apoptosis, signal transduction, immune defense and other physiological processes [43]. Phagocytosis is a basic cellular process that plays an important role in immune system [44]. In this study, RAW 264.7 cells were treated with a series of concentrations of PCP and their effects on NO production and phagocytic activity were investigated. Though the viability of RAW 264.7 cells was not significantly affected in the ranges of investigated concentration of PCP (Fig. 5B), their effects on NO production of macrophages were greatly varied (Fig. 5C). Steaming reduced the effect of PCP on NO production of macrophages. As a result, the effect of PCP0 was the best, while steamed PCP was reduced with increasing steam times, PCP3-PCP4 were only effective at the highest concentration (200 μg/mL), and PCP5 showed no such effect.

Flow cytometry was used to determine the fluorescence intensity in cells after RAW 264.7 cells devoured FITC-dextran. The results showed that LPS (0.4 μg/mL) and PCP could promote the phagocytic activity of macrophages in a dose-dependent manner (Fig. 5D). With the increase of steam times, their ability on phagocytosis weakened, and after the third steam treatment (PCP3-PCP5), they showed no effect on FITC-dextran phagocytose compared with that of blank control group. This variation might attribute to over steaming induced chemical change of active fraction of polysaccharides. However, Wu et al. [17] showed the best activity of *P. cyrtonema* in promoting TNF-α, IL-6 production and NO release at 2 h and 4 h, respectively, of steam treatment. The difference might attribute to different steaming conditions.

## Conclusions

Steaming treatment significantly influenced physicochemical properties and bioactivities of polysaccharides from *P. cyrtonema*, one of the origins of *HuangJing*, a well-known tonic herb. In brief, steaming could significantly increase the molecular weights, UV absorption and antioxidant activity of polysaccharides from *P. cyrtonema*. Polysaccharides with glycosidic linkages such as β-1,4-Gal*p* and β-1,4-man*p* obviously increased after steaming, but fructan could be completely degraded. In addition, steaming could significantly decrease the immunopotentiation activity of polysaccharides from *P. cyrtonema* in certain range. Considering the balance beneficial effects of steaming on antioxidant and immunopotentiation activities of PCP, 2 times of continuous steam is the optimal choice under investigated conditions. However, further study is still necessary to well understand the effect of steaming on *HuangJing*.

## Supplementary Information

**Additional file 1: Table S1.** HPAEC-PAD monosaccharide analysis liquid phase conditions. **Table S2** Molar ratios of compositional monosaccharides for completely acid/pectinase hydrolysates of PCP. **Figure S1. **Ion chromatograms of monosaccharide composition by **(A)** complete acid hydrolysis and **(B)** pectinase hydrolysis. **1–10** in descending order: Fucose, Arabinose, Rhamnose, Galactose, Glucose, Xylose, Mannose, Fructose, Galacturonic Acid, Glucuronic Acid. **Figure S2. **Percentage of compositional monosaccharides molar ratio of **(A)** supernatant alcohol solution, **(B)** complete acid hydrolysate and **(C)** pectinase hydrolysate of PCP0-PCP5. **Figure S3. **The HPSEC-MALLS-RID chromatograms of PCP0-PCP5 after treat with pectinase (PE), β-1,4-Galactanase (GA) and β-1,4-Mannanase (MA). **Figure S4. **HPAEC chromatograms of PCP0-PCP5 after treat with pectinase, β-1,4-Galactanase, β-1,4-Mannanase and TFA. **Figure S5. **The rms conformation plot and conformation plot slope of PCP0-PCP5.

## Data Availability

Data available on request from the authors. The data that support the findings of this study are available from the corresponding author, Shao-ping Li, upon reasonable request.

## Abbreviations

AFM: Atomic force microscopy
ABTS: 2,2'-Azinobis-(3-ethylbenzthiazoline-6-sulphonate)
ANTS: 8-Aminonaphthalene-1,3,6-trisulfonic acid
Ara: Arabinose
CAH: Complete acid hydrolysates
CCK8: Cell counting kit 8
CMM: Chinese Materia medica
DEX: Dextran
DMEM: Dulbecco’s modified eagle medium
DMSO: Dimethyl sulfoxide
dn/dc: Refractive index increment
DPI: Dispersibility index
FBS: Fetal bovine serum
FITC-Dextran: Fluorescein isothiocyanate-dextran
FT-IR: Fourier transform infrared spectroscopy
Fuc: Fucose
Gal: Galactose
GA: Galacturonic acid
GC: Gas chromatography
Glc: Glucose
GlcA: Glucoronic acid
HPAEC: High performance anion exchange chromatography
HPLC: High performance liquid chromatography
HPSEC: High performance size exclusion chromatography
HPTLC: High performance thin layer chromatography
KG: Konjac glucomannan
LPS: Lipopolysaccharides
MALLS: Multi-angle laser light scattering
Man: Mannose
Mw: Molecular weights
Mw/Mn: Polydispersity index
NaOH: Sodium hydroxide
NO: Nitric oxide
P/S: Penicillin/streptomycin
PACE: Carbohydrate gel electrophoresis
PAH: Partial acid hydrolysates
PBS: Phosphate-buffered saline
PC: *Polygonatum cyrtonema*
PCP: *Polygonatum cyrtonema* polysaccharides
PCS: The 75% ethanol supernatant of *Polygonatum cyrtonema* polysaccharides
PGA: Polygalacturonic acid
Rg: Radius of gyration
Rha: Rhamnose
RID: Refractive index detector
SEC: Size exclusion chromatography
SEM: Standard error of mean
TCM: Traditional Chinese medicine
TFA: Trifluoroacetic acid
TLC: Thin layer chromatography
UV: Ultraviolet
Vc: Ascorbic acid
Xyl: Xylose
