# Supplementary material for: Effects of steam on polysaccharides from Polygonatum cyrtonema based on saccharide mapping analysis and pharmacological activity assays
Source: Chin Med. 2022 Aug 17;17:97. doi: 10.1186/s13020-022-00650-3 (PMC9386940; doi:10.1186/s13020-022-00650-3)
Supplement: Supplementary file 1 — Additional file 1: Table S1. HPAEC-PAD monosaccharide analysis liquid phase conditions. Table S2 Molar ratios of compositional monosaccharides for completely acid/pectinase hydrolysates of PCP. Figure S1. Ion chromatograms of monosaccharide composition by (A) complete acid hydrolysis and (B) pectinase hydrolysis. 1–10 in descending order: Fucose, Arabinose, Rhamnose, Galactose, Glucose, Xylose, Mannose, Fructose, Galacturonic Acid, Glucuronic Acid. Figure S2. Percentage of compositional monosaccharides molar ratio of (A) supernatant alcohol solution, (B) complete acid hydrolysate and (C) pectinase hydrolysate of PCP0-PCP5. Figure S3. The HPSEC-MALLS-RID chromatograms of PCP0-PCP5 after treat with pectinase (PE), β-1,4-Galactanase (GA) and β-1,4-Mannanase (MA). Figure S4. HPAEC chromatograms of PCP0-PCP5 after treat with pectinase, β-1,4-Galactanase, β-1,4-Mannanase and TFA. Figure S5. The rms conformation plot and conformation plot slope of PCP0-PCP5. [file 13020_2022_650_MOESM1_ESM.docx]

**Effects of steam on polysaccharides from *Polygonatum cyrtonema* based on** **saccharide mapping analysis and pharmacological activity assays**

Zherui Chen **^a,b^**, Baojie Zhu **^a,b^**, Jing Zhao ^a,b*^, Zhixin Chen **^a,b^**, Wen Cao **^a,b^**, Junqiao Wang **^a,b^**, Shaoping Li ^a,b*^

**a** State Key Laboratory of Quality Research in Chinese Medicine, Institute of Chinese Medical Sciences, University of Macau, Macao SAR, China

**b** Joint Laboratory of Chinese Herbal Glycoengineering and Testing Technology, University of Macau,
Macao SAR, China

***Corresponding authors:**

**Zhao Jing**

Email: [jingzhao@um.edu.mo](mailto:jingzhao@um.edu.mo) (J Zhao); [spli@um.edu.mo](mailto:spli@um.edu.mo) or [lishaoping@hotmail.com](mailto:lishaoping@hotmail.com) (SP Li)

| **Table S1** HPAEC-PAD monosaccharide analysis liquid phase conditions | | | | | |
| --- | --- | --- | --- | --- | --- |
| Time(min) | Flow(ml/min) | A% | B% | C% | D% |
| -31 | 0.4 | 88 | 12 | 0 | 0 |
| -21 | 0.4 | 88 | 12 | 0 | 0 |
| -20 | 0.4 | 0 | 0 | 100 | 0 |
| -11 | 0.4 | 0 | 0 | 100 | 0 |
| -10 | 0.4 | 88 | 12 | 0 | 0 |
| 0 | 0.4 | 88 | 12 | 0 | 0 |
| 10 | 0.4 | 88 | 12 | 0 | 0 |
| 22 | 0.4 | 43 | 12 | 0 | 45 |
| 25 | 0.4 | 43 | 12 | 0 | 45 |
| 26 | 0.4 | 88 | 12 | 0 | 0 |
| A：ddwater；B：NaOH（10 mM）；C：NaOH（200mM）；D：NaOAc（0.5 M）； | | | | | |

| **Table S2** Molar ratios of compositional monosaccharides for completely acid/pectinase hydrolysates of PCP | | | | | | | | |
| --- | --- | --- | --- | --- | --- | --- | --- | --- |
| Sample | Ara | Rha | Gal | Glc | Xyl | Man | Fru | GalA |
| PCP0 | 21.0/59.4 | 6.7/7.5 | 18.3/56.3 | 56.3/57.5 | 1.0 | 107.4/59.7 | 14.3/415.2 | 41.1/263.0 |
| PCP1 | 9.4/33.5 | 4.0/8.9 | 29.2/102.7 | 4.2/5.8 | 1.0 | 24.0/22.9 | 0.1/6.8 | 20.0/150.5 |
| PCP2 | 7.7/26.4 | 4.9/9.8 | 38.5/137.5 | 3.2/6.4 | 1.0 | 20.8/21.0 | 0.1/2.5 | 15.2/105.1 |
| PCP3 | 4.5/12.4 | 5.6/10.9 | 41.6/120.5 | 2.5/6.7 | 1.0 | 15.0/16.5 | 0.1/1.8 | 14.6/84.6 |
| PCP4 | 2.7/7.6 | 4.9/10.3 | 32.5/98.7 | 2.2/5.7 | 1.0 | 12.6/18.0 | 0.0/1.6 | 10.6/63.8 |
| PCP5 | 1.6/3.7 | 5.0/10.4 | 29.3/85.9 | 2.3/3.6 | 1.0 | 12.7/18.8 | 0.0/0.8 | 8.5/47.3 |


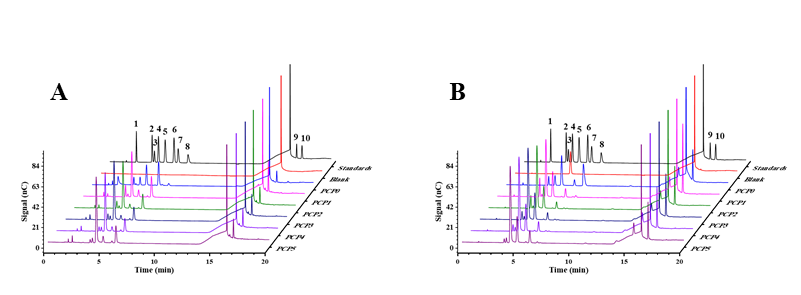


**Figure S1.** Ion chromatograms of monosaccharide composition by **(A)** complete acid hydrolysis and **(B)** pectinase hydrolysis. **1–10** in descending order: Fucose, Arabinose, Rhamnose, Galactose, Glucose, Xylose, Mannose, Fructose, Galacturonic Acid, Glucuronic Acid.


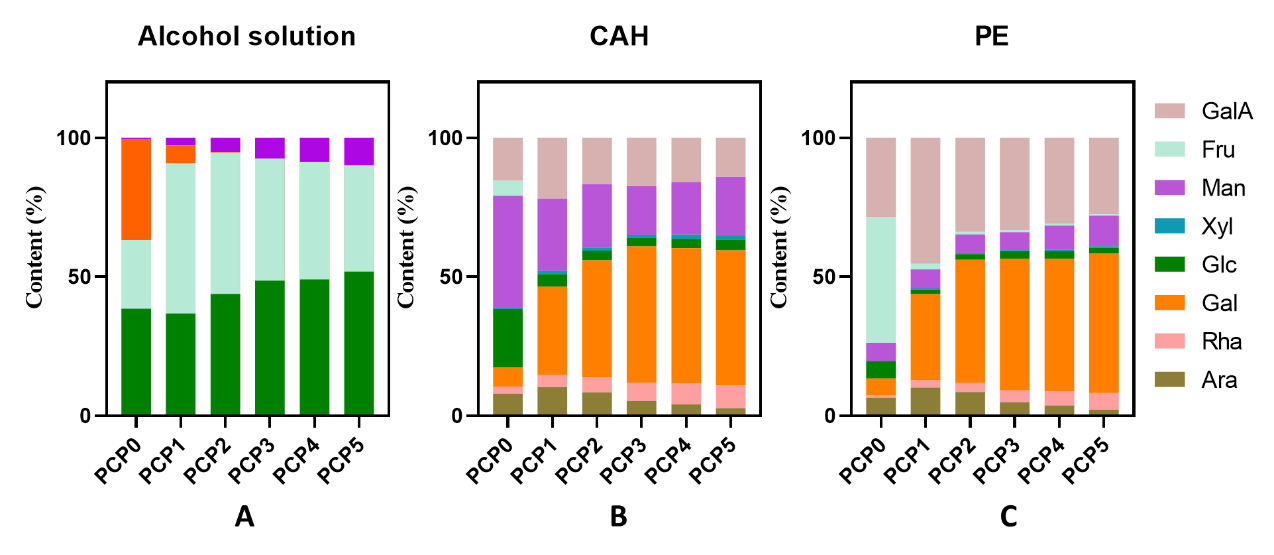


**Figure S2.** Percentage of compositional monosaccharides molar ratio of **(A)** supernatant alcohol solution, **(B)** complete acid hydrolysate and **(C)** pectinase hydrolysate of PCP0-PCP5.


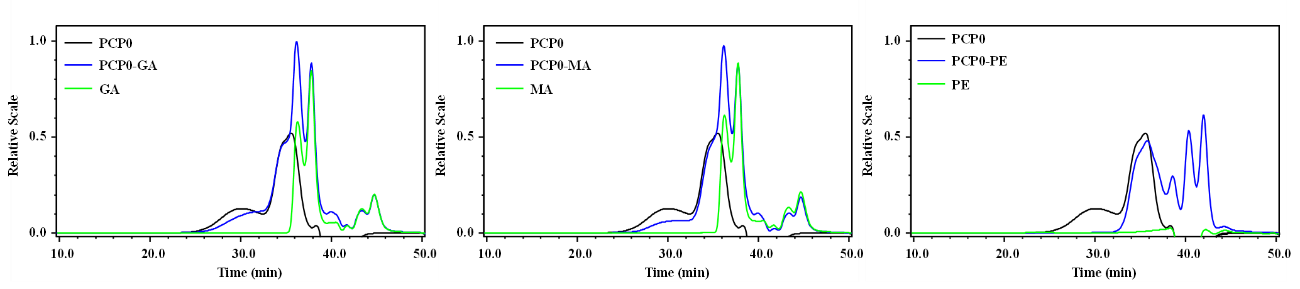


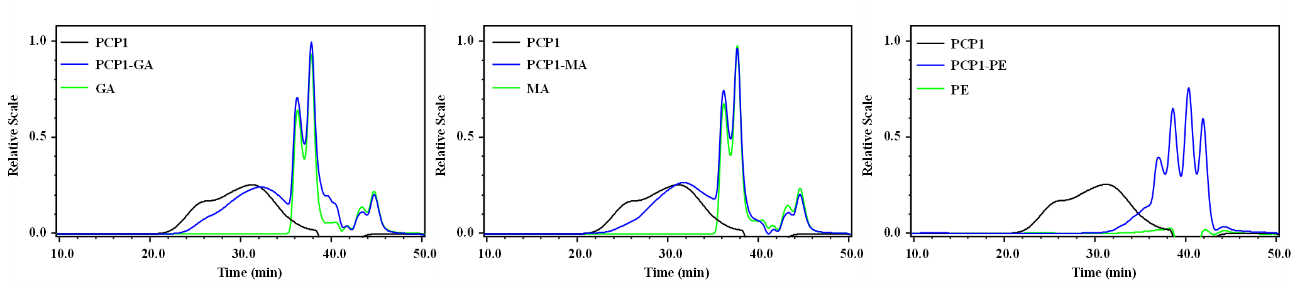


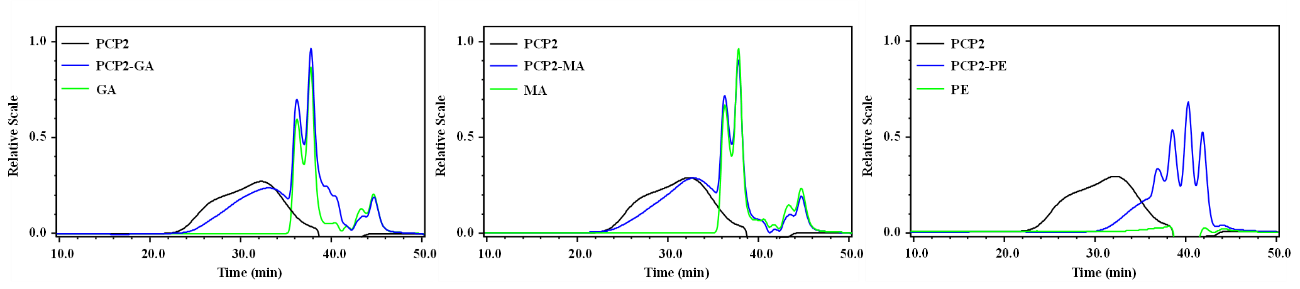


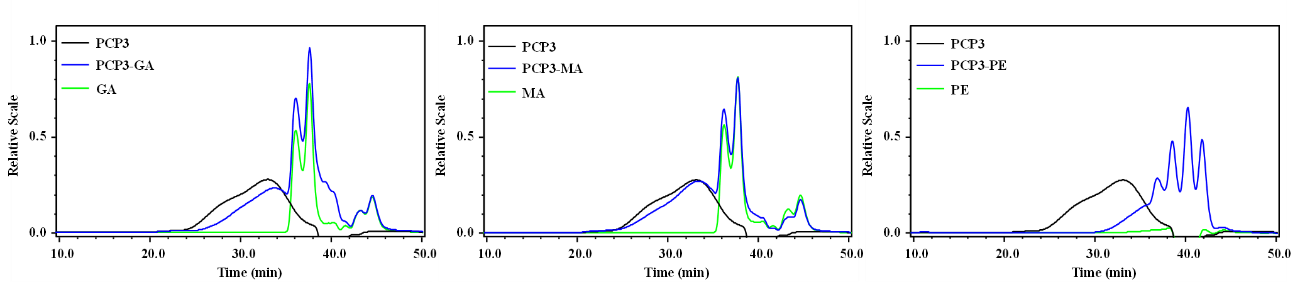


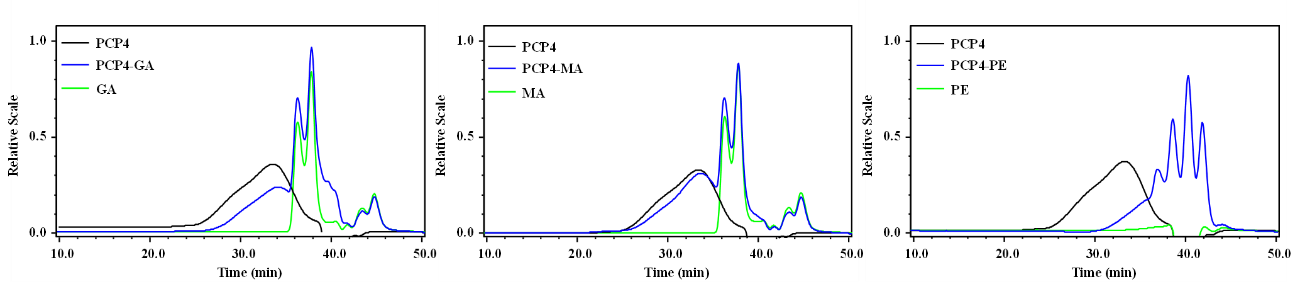


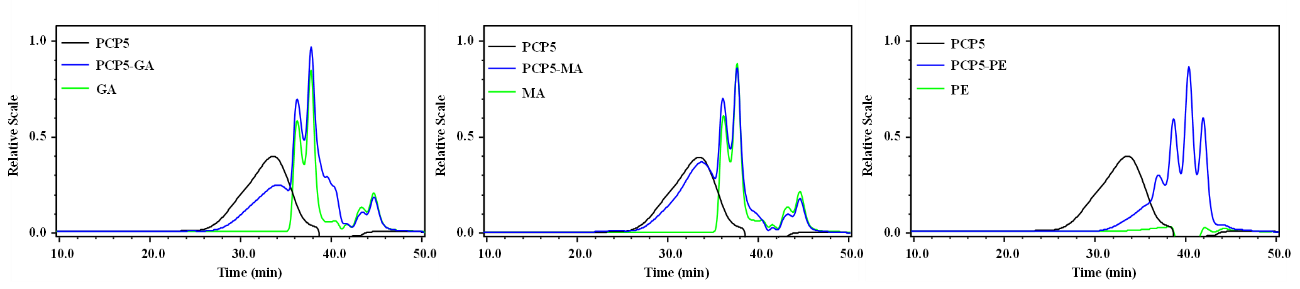


**Figure S3.** The HPSEC-MALLS-RID chromatograms of PCP0-PCP5 after treat with pectinase (PE), β-1,4-Galactanase (GA) and β-1,4-Mannanase (MA).


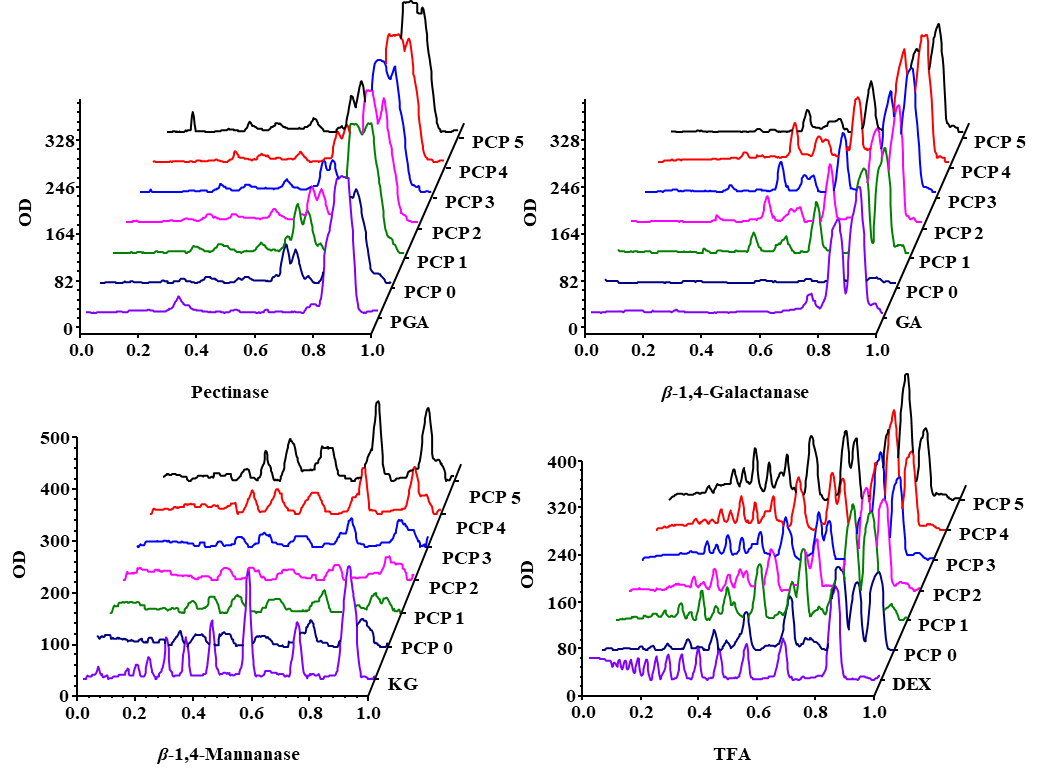


**Figure S4.** HPAEC chromatograms of PCP0-PCP5 after treat with pectinase, β-1,4-Galactanase, β-1,4-Mannanase and TFA.

PGA, Polygalacturonic Acid; GA, Galacturonic Acid; KG, Konjac Glucomannan; DEX, Dextran.

**
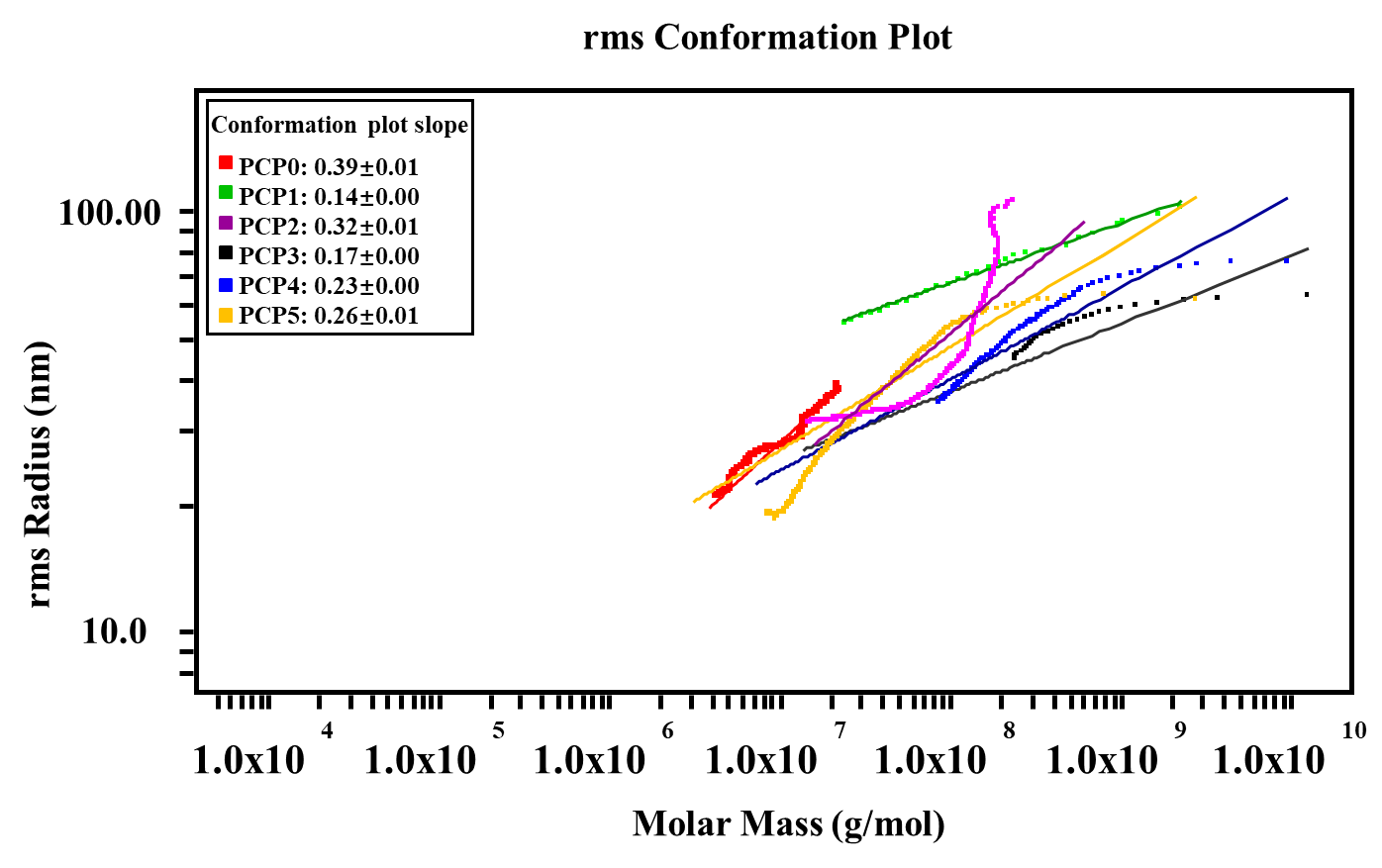
**

**Figure S5.** The rms conformation plot and conformation plot slope of PCP0-PCP5.
